# Efficacy of Artesunate-mefloquine for Chloroquine-resistant *Plasmodium vivax* Malaria in Malaysia: An Open-label, Randomized, Controlled Trial

**DOI:** 10.1093/cid/ciw121

**Published:** 2016-05-12

**Authors:** Matthew J. Grigg, Timothy William, Jayaram Menon, Bridget E. Barber, Christopher S. Wilkes, Giri S. Rajahram, Michael D. Edstein, Sarah Auburn, Ric N. Price, Tsin W. Yeo, Nicholas M. Anstey

**Affiliations:** 1 Global and Tropical Health Division, Menzies School of Health Research and Charles Darwin University, Darwin, Northern Territory, Australia; 2 Infectious Diseases Society Sabah-Menzies School of Health Research Clinical Research Unit, Kota Kinabalu; 3 Clinical Research Centre, Queen Elizabeth Hospital; 4 Jesselton Medical Centre; 5 Sabah Department of Health, Kota Kinabalu, Malaysia; 6 Department of Drug Evaluation, Australian Army Malaria Institute, Brisbane, Queensland; 7 Centre for Tropical Medicine and Global Health, Nuffield Department of Clinical Medicine, University of Oxford, United Kingdom; 8 Lee Kong Chian School of Medicine, Nanyang Technological University, Singapore; 9 Division of Medicine, Royal Darwin Hospital, Darwin, Northern Territory, Australia

**Keywords:** *Plasmodium vivax*, malaria, randomized, controlled trial, artesunate-mefloquine, chloroquine

## Abstract

***Background.*** Chloroquine (CQ)-resistant *Plasmodium vivax* is increasingly reported throughout southeast Asia. The efficacy of CQ and alternative artemisinin combination therapies (ACTs) for vivax malaria in Malaysia is unknown.

***Methods.*** A randomized, controlled trial of CQ vs artesunate-mefloquine (AS-MQ) for uncomplicated vivax malaria was conducted in 3 district hospitals in Sabah, Malaysia. Primaquine was administered on day 28. The primary outcome was the cumulative risk of treatment failure by day 28 by Kaplan–Meier analysis.

***Results.*** From 2012 to 2014, 103 adults and children were enrolled. Treatment failure by day 28 was 61.1% (95% confidence interval [CI], 46.8–75.6) after CQ and 0% (95% CI, 0–.08) following AS-MQ (*P* < .001), of which 8.2% (95% CI, 2.5–9.6) were early treatment failures. All patients with treatment failure had therapeutic plasma CQ concentrations at day 7. Compared with CQ, AS-MQ was associated with faster parasite clearance (normalized clearance slope, 0.311 vs 0.127; *P* < .001) and fever clearance (mean, 19.0 vs 37.7 hours; *P =* .001) and with lower risk of anemia at day 28 (odds ratio = 3.7; 95% CI, 1.5–9.3; *P =* .005). Gametocytes were present at day 28 in 23.8% (10/42) of patients following CQ vs none with AS-MQ (*P* < .001). AS-MQ resulted in lower bed occupancy: 4037 vs 6510 days/1000 patients (incidence rate ratio 0.62; 95% CI, .60–.65; *P* < .001). One patient developed severe anemia not regarded as related to their AS-MQ treatment.

***Conclusions.*** High-grade CQ-resistant *P. vivax* is prevalent in eastern Malaysia. AS-MQ is an efficacious ACT for all malaria species. Wider CQ-efficacy surveillance is needed in vivax-endemic regions with earlier replacement with ACT when treatment failure is detected.

**Clinical Trials Registration**. NCT01708876.

## Abstract (precis)

High-grade chloroquine (CQ)-resistant *Plasmodium vivax* is prevalent in eastern Malaysia. Artesunate-mefloquine is an efficacious artemisinin combination therapy (ACT) for all malaria species. Wider CQ-efficacy surveillance is needed in vivax-endemic regions with earlier replacement with ACT when treatment failure is detected.

The global population at risk of malaria due to infection with *Plasmodium vivax* is estimated at 2.48 billion people [1], with southeast Asia accounting for up to 67% of symptomatic cases [2]. *Plasmodium vivax* causes substantial morbidity due to recurrent infections [3], which are associated with anemia [4], adverse pregnancy outcomes [5], and ability to cause severe disease and death [6–8]. While malaria control programs have been highly effective in reducing the incidence of vivax malaria in Malaysia [9, 10], transmission persists in rural areas, including the likelihood of a significant reservoir of asymptomatic or submicroscopic carriage [11]. A recent population genetic analysis of *P. vivax* in Sabah, Malaysia, was consistent with low endemicity and multiple subpopulations, resulting in vulnerability to epidemic expansions from new or introduced parasite strains [12].

Current Malaysian Ministry of Health malaria treatment guidelines recommend chloroquine (CQ) and primaquine for uncomplicated vivax malaria [13]. CQ-resistant *P. vivax* in the presence of therapeutic CQ concentrations has been documented in a returned traveller from Sabah in 1996 [14] and has been suggested by retrospective hospital-based studies from peninsular Malaysia [15, 16], but as yet no systematic clinical evaluation has been undertaken. Malaysia is located in close geographical proximity to countries with confirmed CQ-resistant *P. vivax*, including high-grade CQ-resistance in Indonesia and Papua New Guinea and low-grade CQ-resistance in Philippines, Thailand, and Vietnam [17]. The area is vulnerable to importation of CQ resistance from a large transient population that includes migrant workers. Due to the lack of reliable molecular markers of CQ-resistant *P. vivax*, ongoing clinical monitoring of antimalarial efficacy is vital to guide effective *P. vivax* treatment policy [18], particularly in Malaysia, which aims to eliminate malaria by 2020 [9].

There is growing support for artemisinin-based combination therapy (ACT) as a unified first-line treatment in areas co-endemic for *Plasmodium falciparum* and *P. vivax* [19]. In Sabah *Plasmodium knowlesi* is also prevalent [10], with frequent microscopic misdiagnoses between *P. vivax*, *P. knowlesi* and *P. falciparum* [20]. A unified policy of ACT would improve the early therapeutic response, reduce the risk of anemia, and decrease healthcare costs compared with a policy of CQ for uncomplicated nonfalciparum malaria [19, 21]; furthermore such a policy would reduce morbidity and mortality associated with the inadvertent use of CQ for CQ-resistant *P. falciparum*.

Artesunate-mefloquine (AS-MQ) is 1 of 2 ACTs recommended for *P. falciparum* and *P. knowlesi* malaria in Malaysia [13]. Our aim in this study was to assess the presence of CQ-resistant *P. vivax* and determine whether the fixed combination of AS-MQ was superior to CQ for blood-stage treatment of uncomplicated vivax malaria in Malaysia.

This study was approved by the relevant human medical research ethics committees of the Malaysian Ministry of Health, and Menzies School of Health Research, Australia. Approval for drug analysis was obtained from the Australian Defence Human Research Ethics Committee (ADHREC 717-13).

## METHODS

This 2-arm, randomized, open-label trial was conducted at 3 hospitals in Sabah: Kudat, Kota Marudu, and Pitas District. Included were patients with acute, uncomplicated vivax malaria presenting to the study hospitals, aged >1 year and weighing >10 kg, with microscopic diagnosis of *P. vivax* monoinfection, negative for *P. falciparum* by rapid diagnostic test (histidine-rich protein-2), and fever (≥37.5°C) or history of fever in the last 48 hours. Written informed consent was obtained from the patient or their guardian. Exclusion criteria were severe malaria or warning signs according to modified World Health Organization (WHO) 2010 criteria [7], parasitemia >20 000/μL until March 2013 and >100 000/μL thereafter, pregnancy or lactation, known hypersensitivity or contraindication to any study drug, any serious underlying medical condition, or antimalarial use in the previous 2 months.

### Treatment Interventions

AS-MQ (Mepha, Switzerland) was administered as a fixed-dose combination with 3 oral formulations (600/1500 mg, 300/750 mg, or 50/125 mg). Doses were administered at enrollment and 24 and 48 hours after (target total dose 12 mg/kg AS and 25 mg/kg MQ). CQ diphosphate (Kotra Pharma Sdn Bhd, Malaysia), consisting of 155 mg base tablets, was administered at enrollment and 6, 24, and 48 hours after (target total dose 25 mg/kg). Primaquine (Pharmaniaga Sdn Bhd, Malaysia) as 15-mg tablets was commenced on day 28 (per WHO treatment surveillance guidelines in order not to underestimate the true risk of CQ resistance with concomitant primaquine [22]) to glucose-6-phosphate dehydrogenase (G6PD)–normal patients as 30 mg given daily or, if weighing ≤35 kg, 0.5 mg/kg daily for 14 days. Dosages of all study drugs were based on body weight [13, 23]. Study drugs were certified under Good Manufacturing Practices. Drug administration was supervised by a study team member, and patients were observed for 1 hour post-treatment with readministration if vomiting occurred.

### Outcomes and Power Calculation

The primary endpoint was the cumulative risk of treatment failure by day 28 as defined by WHO [22]. Secondary endpoints included early and late treatment failures (LTF) [22]; treatment failure risk by day 42; the proportion of patients aparasitemic at 24, 48, and 72 hours; parasite and fever clearance times; the linear slope constant of the log*_e_* parasite-time profile [24]; the risk of anemia according to WHO age-based criteria at day 28 [25]; the fractional fall in hemoglobin at day 3; the nadir hemoglobin concentration; the proportion with *P. vivax* gametocytes at day 28; the risk of adverse events; and the length of hospitalization. A total of 66 patients in each arm were required to detect an absolute difference of 15% in day 28 treatment failure rates between the study arms, with 80% power and 95% confidence, assuming a CQ efficacy of 85% and 10% loss to follow-up.

### Randomization and Blinding

Patient allocation codes were computer generated in blocks of 20 for each study arm using STATA (version 12) by an independent statistician, separately sealed in an opaque envelope, and opened by a study nurse after patient enrollment. The primary endpoint and other parasitological measurements were determined by microscopists blinded to patient treatment allocation.

### Study Procedures

Polymerase chain reaction (PCR) confirmation of species [26] and G6PD activity (Beutler fluorescent spot test) were assessed from venous blood taken at enrollment. All patients had 6-hourly temperature measurements and finger-prick blood sampling to quantify fever and parasite clearance, respectively. Symptoms were recorded daily in a standard questionnaire and the risks of adverse event determined. Patients were followed up as outpatients on days 7, 14, 28, and 42 after enrollment, and blood was taken for smear examination and hemoglobin concentrations. Patients who met criteria for treatment failure were administered rescue medication with the alternative study drug and primaquine.

### Genotyping

*Plasmodium vivax* genotyping was performed with 9 previously described short tandem repeat markers: *Pv3.27, msp1F3, MS1, MS5, MS8, MS10, MS12, MS16*, and *MS20* (see Supplementary Material) [27, 28].

### Chloroquine and Desethylchloroquine Concentrations

Heparinized blood samples were collected on day 7 [29, 30] and the day of recurrent parasitemia [22]. Blood samples were centrifuged and plasma separated and stored at −20°C until analyzed. CQ and its major active metabolite desethylchloroquine (DCQ) were assayed by liquid chromatography-tandem mass spectrometry (see Supplementary Material). CQ resistance was defined as failure to clear peripheral *P. vivax* parasitemia on blood smear or recurrent parasitemia within 42 days in the presence of plasma blood concentration of CQ + DCQ in excess of the minimal effective concentration (MEC > 15 ng/mL CQ + DCQ), corresponding to a whole blood concentration of 100 ng/mL [22, 31]. Patients were characterized as having high-grade CQ resistance if there was early treatment failure with plasma concentration greater than the MEC at day 7.

### Statistical Methods

Data were double-entered into Epidata (version 3.1) and analyzed with STATA (version 12). Primary analysis was intention-to-treat, with incidence risk of treatment failure at days 28 and 42 calculated using the Kaplan–Meier method and compared using the Mantel-Haenszel log-rank test. Intergroup differences were compared using the Student *t* test or Wilcoxon-Mann–Whitney test for continuous variables and using χ^2^ or Fisher exact test for categorical variables. Microscopic asexual parasitemia and gametocytemia were calculated from thick blood smears [32]. Best-fit linear or tobit polynomial regression models were used to estimate the curve of log*_e_* parasite counts vs time per the parasite clearance methodology of the World Wide Antimalarial Resistance Network [24].

## RESULTS

**Figure 1.**
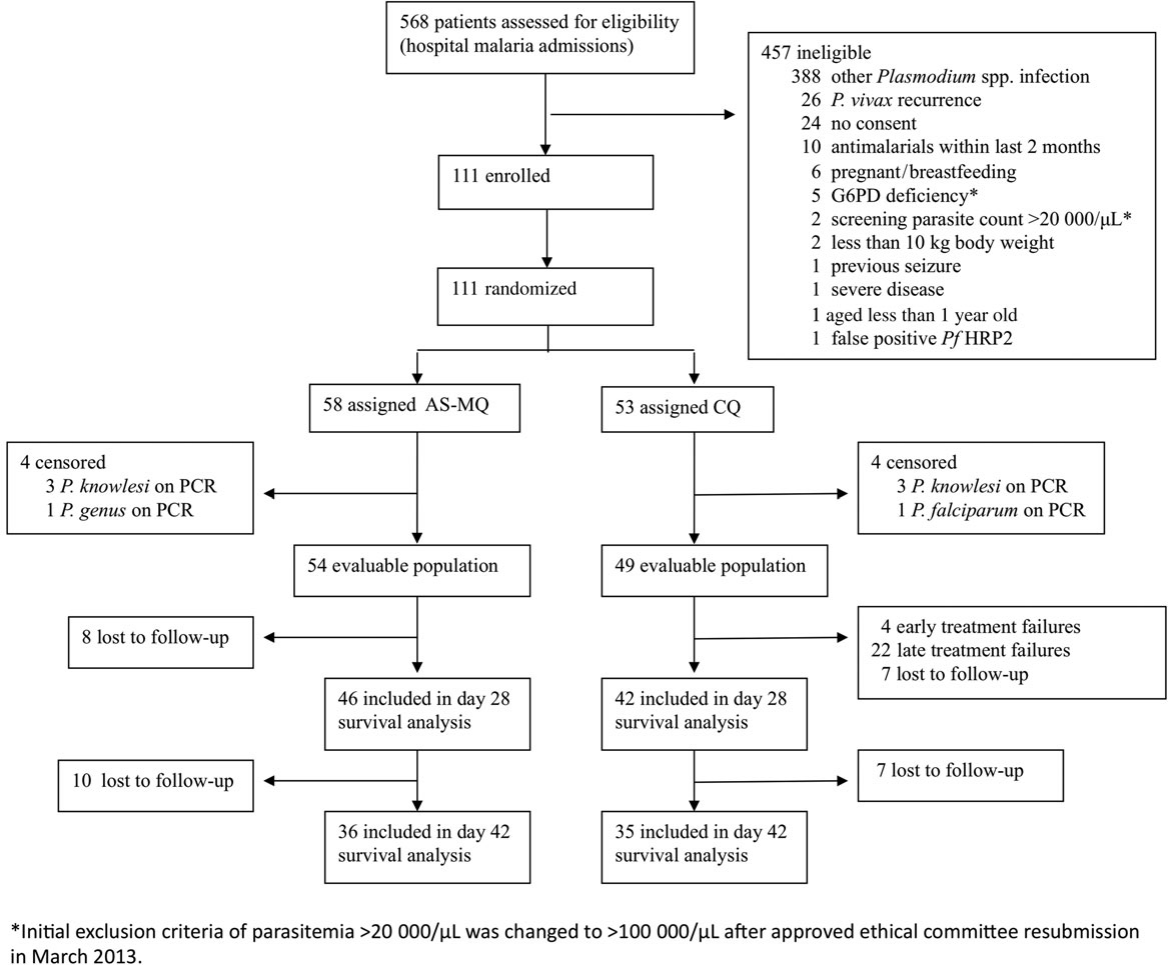
Study profile. Abbreviations: AS-MQ, artesunate-mefloquine; CQ, chloroquine; PCR, polymerase chain reaction.

Between October 2012 and December 2014, 33.5% (190/568) of malaria patients were diagnosed with *P. vivax* monoinfection, of whom 58.4% (111/190) were enrolled in the study (Figure 1). Eight patients initially randomized were subsequently censored from analysis due to PCR diagnosis of other *Plasmodium* spp., with 88 patients completing follow-up to day 28.

In view of the high risk of treatment failure in the CQ treatment arm, an independent interim analysis was undertaken following which the study was stopped due to the large difference in the primary outcome between treatment arms.

### Baseline Data

There were no differences in baseline patient demographics between treatment groups (Table 1). The median age of patients was 17 years (range 1–65), with 37.9% (39/103) aged <12 years. The geometric mean parasitemia at presentation was 4022 parasites/μL in the AS-MQ arm and 3082 parasites/μL in the CQ arm. Blood cultures were taken in 81.6% (84/103), with no clinically significant pathogens isolated. Patients completing CQ treatment received a median total of 27.7 mg/kg (range, 25.0–33.0) of CQ, and those administered AS-MQ received a median 11.0 mg/kg (range, 9.8–12.0) AS and 27.6 mg/kg (range, 24.6–30.0) MQ. Two patients in the CQ arm required readministration of the drug within 1 hour after vomiting.

**Table 1.** Demographic and Baseline Characteristics

Variable	Artesunate-Mefloquine n = 54	Chloroquine n = 49
Age		
median	17	17
(IQR) [range]	(9–31)[1–65]	(10–34)[3–57]
Age ≤ 12 y		
n (%)	21 (38.9)	18 (36.7)
Gender		
n male (%)	33 (61.1)	33 (67.3)
Body weight		
median kg (IQR)	42 (24–57)	44 (25–56)
Fever ( ≥ 37.5°C) on admission		
n (%)	28 (51.9)	23 (46.9)
Fever history duration		
median days	5	5
(IQR) [range]	(3–7)[0–14]	(4–7)[1–14]
Parasite count/μL		
geometric mean	4022	3082
(95% confidence interval)	2955–5474	2223–4272
median	5280	3131
(IQR) [range]	(1908–9608)[174–20064]	(1584–6350)[240–42521]
Gametocytes present		
n (%)	35 (65)	22 (49)
Hemoglobin		
median g/dL	12.2	12.4
(IQR) [range]	(10.4–13.3)[7.2–16.6]	(10.1–13.9)[8.3–16.1]

Abbreviation: IQR, interquartile range.

### Early Therapeutic Response

Parasite clearance times (PCT) were significantly faster with AS-MQ (median, 19.7 hours; interquartile range [IQR], 18.0–26.4) compared with CQ (median, 48 hours; IQR, 30–54); *P <* .001), and this was reflected by the slope of the log_10_ normalized parasite clearance curve (0.311 vs 0.127; *P <* .001; Table 2). At 72 hours post-treatment, 16.3% (8/49) of participants in the CQ arm were parasitemic compared with none in the AS-MQ arm (*P =* .002). Clearance was faster after AS-MQ than CQ for both ring-stage (mean time to 50% clearance [PCT_50_], 7.3 vs 12.9 hours, respectively; *P* = .005) and the trophozoite-stages (PCT_50_, 11.3 vs 27.9 hours following CQ; *P <* .001; Table 2). Patients with slow parasite clearance (>48 hours) after CQ were more likely to have recurrent parasitemia by day 28 (odds ratio [OR], 11.2; 95% confidence interval [CI], 2.1–60.0; *P* = .005). Fever clearance time correlated with PCT (r_s_ = 0.449; *P =* .001) and was faster with AS-MQ (19.0 hours; IQR, 10.6–27.4) compared with CQ (37.7 hours; IQR, 26.6–48.7; *P =* .001). The median number of days spent in the hospital, including readmission for recurrent parasitemia, was higher in the CQ arm (5 days; IQR, 4–6) vs the AS-MQ arm (4 days; IQR, 3–4; *P =* .001), the cumulative bed occupancy being 6510 days/1000 persons and 4037 days/1000 persons after CQ and AS-MQ treatment, respectively (incidence rate ratio, 0.62; 95% CI, .60–.65; *P* < .001).

**Table 2.** Parasite Clearance

Variable	Artesunate-Mefloquine n = 54	Chloroquine n = 49	*P* Value
Parasite clearance			
24 h			
n (%)	42 (77.8)	7 (14.3)	**<.001**
95% CI	64.4–88.0	5.9–27.2	
HR (95% CI)	21.0 (7.5–58.6)		
48 h			
n (%)	52 (96.3)	28 (57.1)	**<.001**
95% CI	91.1–100	42.2–71.2	
HR (95% CI)	19.5 (4.3–89.3)		
72 h			
n (%)	54 (100)	41 (83.7)	**.002**
95% CI	93.4–100	72.9–94.4	
Parasite clearance time^a^			
median hours	19.7	48	**<.001**
(IQR) [range]	(18–26.1)[7.7–72]	(30–54)[12–90]	
Slope of curve *(k)* for log_10_			
normalized parasite clearance			
mean *(k)* constant	0.311	0.127	**<.001**
95% CI	0.277–0.346	0.108–0.147	
Lag phase present			
n (%)	0	11 (22)	**.016**
PCT_50_			
mean hours (95% CI)	7.1 (6.6–7.7)	12.0 (9.8–14.2)	**<.001**
PCT_90_			
mean hours (95% CI)	10.1 (9.2–11.1)	26.0 (21.1–30.9)	**<.001**
PCT_95_			
mean hours (95% CI)	12.0 (10.6–13.4)	31.5 (26.5–36.5)	**<.001**
PCT_99_			
mean hours (95% CI)	16.8 (14.6–18.9)	39.1 (33.9–44.2)	**<.001**
Gametocyte clearance time			
mean hours (95% CI)	9.4 (6.5–12.4)	16.4 (9.5–23.3)	.834
Gametocytes			
Positive day 7 n (%)	0/43 (0)	1/41 (2.4)	.488
Positive day 28	0/46 (0)	10/42 (23.8)	**<.001**

Bold was used to highlight statistically significant values (*P* < .05).

Abbreviations: CI, confidence interval; HR, hazard ratio; IQR, interquartile range; PCT, parasite clearance time.

^a^ Censored from PCT analysis were patients meeting criteria for early treatment failure (ETF) in addition to 4 late treatment failure patients (not meeting ETF criteria) with cross-check research microscopy still positive for *Plasmodium vivax* parasites at time of hospital discharge.

### Treatment Outcomes

**Figure 2.**
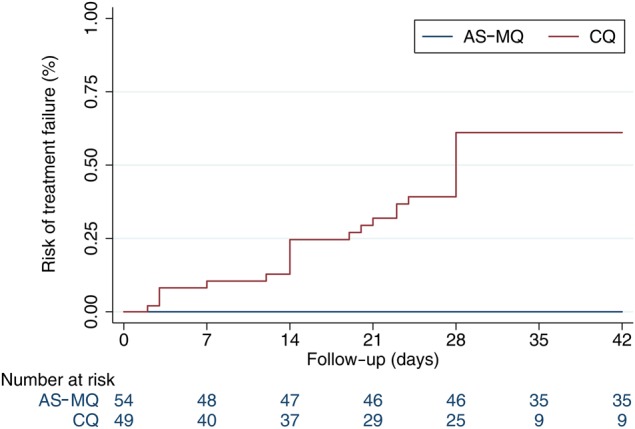
Kaplan–Meier treatment failure estimates. Abbreviations: AS-MQ, artesunate-mefloquine; CQ, chloroquine.

By day 28 the cumulative risk of treatment failure was 61.1% (95% CI, 46.8–75.6; 26/42) in the CQ arm compared with 0% (0/46) in the AS-MQ arm (*P* < .001; Figure 2 and Table 3). Of patients treated with CQ, 8.2% (4/49) and 52.4% (22/42) experienced early treatment failures and LTF, respectively (Table 3). All patients remaining aparasitemic at day 28 commenced primaquine. No patients developed recurrent parasitemia between days 28 and 42 of follow-up. In the AS-MQ arm no patients were gametocytemic by day 28 compared with 23.8% (10/42) of those in the CQ arm (*P* < .001). Of the 26 patients with CQ treatment failure treated with AS-MQ, 76.9% (20) could be followed up for an additional 28-day period from the date of administration, none of whom had further recurrence.

**Table 3.** Treatment Outcomes

Variable	Artesunate-Mefloquine n = 54	Chloroquine n = 49	*P* Value
Treatment failure by day 28^a^			
Kaplan–Meier estimates % risk	0	61.1	<.001
95% confidence interval	0–7.7	46.8–75.6	
n/N	0/46	26/42	
Early treatment failure			
n/N (%)	0	4/49 (8.2)	<.001
Late treatment failure			
Total n/N (%)	0	22/42 (52.4)	<.001
Clinical n/N (%)	…	15/42 (35.7)	
Parasitological n/N (%)	…	7/42 (16.7)	
Day of recurrence^b^			
median	…	23	…
(interquartile range) [range]	…	(14–28)[7–28]	

^a^ There were no additional treatment failures after day 28; therefore, Kaplan–Meier estimates at day 42 were the same as at day 28.

^b^ Late treatment failure only.

### Hematological Outcomes

**Figure 3.**
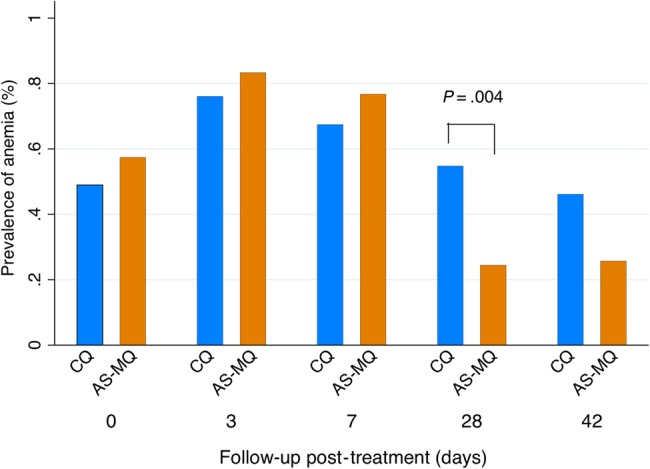
Anemia prevalence. Abbreviations: AS-MQ, artesunate-mefloquine; CQ, chloroquine.

By day 28, 54.8% (23/42) of patients treated with CQ were anemic compared with 24.4% (11/45) treated with AS-MQ (OR = 3.7; 95% CI, 1.5–9.3; *P =* .005; Table 4 and Figure 3). This difference remained after adjusting for confounding factors including age, sex, and baseline parasitemia and hemoglobin (adjusted OR, 6.8; 95% CI, 2.1–22.2; *P =* .001). There were no differences in any of the other secondary hematological outcomes. One adult patient in the AS-MQ arm developed severe anemia (hemoglobin <7 g/dL [33]) on day 3.

**Table 4.** Hematological Outcomes

Variable	Artesunate-Mefloquine n = 54	Chloroquine n = 49	*P* Value
Fractional fall in hemoglobin at day 3			
mean % fall	12.6	13.2	.682
95% CI	10.9–14.3	10.9–15.4	
Hemoglobin nadir (g/dL)			
median	10.5	10.3	.840
IQR (range)	9.3–11.3 (6.7–14.1)	9–11.8 (6.4–13.2)	
Time to hemoglobin nadir (days)			
median	2	2	.942
IQR (range)	2–3 (0–42)	1–4 (0–28)	
Prevalence of anemia at day 28			
%	24.4	54.8	**.004**
95% CI	12.9–39.5	38.7–70.2	
n/N	11/45	23/42	
AOR^a^ (95% CI)		6.8 (2.1–22.0)	.001
Prevalence of anemia at day 42^b^			
%	25.7	53.3	**.023**
95% CI	12.5–43.3	34.3–71.7	
n/N	9/35	16/30	
AOR^a^ (95% CI)		3.7 (1.0–13.5)	.045

Bold was used to highlight statistically significant values (*P* < .05).

Abbreviations: AOR, adjusted odds ratio; CI, confidence interval; IQR, interquartile range.

^a^ After controlling for the following confounding factors: baseline parasite count, hemoglobin, age, sex.

^b^ Post primaquine administration.

### *Plasmodium* vivax Genotyping and PCR Adjustment

Blood samples were available from day 0 and the day of failure for 77% (17/22) of patients with LTF. PCR amplification/genotyping was successful in all samples analyzed. Comparison of the genotype profiles between day 0 and recurrent infections revealed that all 17 recurrences were homologous. The PCR-adjusted cure rate was therefore identical to the clinical/parasitological cure rate.

### Plasma Chloroquine and Desethylchloroquine Concentrations

Blood samples were obtained from 22 (88%) of the 25 patients with treatment failure by day 28 who completed a full course of CQ. Of the 16 patients with no treatment failure, blood samples were obtained from 15 (94%). All patients had therapeutic plasma CQ concentrations at day 7 post-treatment. Of those with recurrent parasitemia, 45% (9/20) had composite plasma CQ and DCQ concentrations greater than the MEC of 15 ng/mL on the day of recurrence, while those below this cutoff presented later (median 28 days; IQR, 21–28 vs 12 days; IQR, 3–20; *P* = .0013) (see Supplementary Material).

### Adverse Events

One serious adverse event was reported in the AS-MQ arm: severe anemia 3 days after commencing treatment, not thought to be related to the study medication. Other adverse events between treatment groups were comparable, including the proportion with headache (91%), dizziness (77%), vomiting (51%), or hearing changes potentially associated with MQ use, or in grouped-system analyses (see Supplementary Material).

## DISCUSSION

This study highlights the presence of high-grade CQ-resistant *P. vivax* infections in Sabah, eastern Malaysia. The large proportion of patients who failed treatment with CQ by day 28 is comparable to that seen in neighboring Kalimantan, Indonesian Borneo [34], and approaches that seen in Papua, Indonesia [35]. CQ-resistant *P. vivax* strains may have arisen de novo in the local population as a result of longstanding pressure to use CQ in Malaysia or may have been introduced from neighboring areas due to migration or vector spread [12]. In contrast, AS-MQ was highly efficacious in this region of CQ-resistant *P. vivax*, with adequate parasitological and clinical cure in all patients followed up to day 42 post-treatment.

The efficacy of AS-MQ against *P. vivax* is consistent with that found in previous clinical trials of ACTs with long-acting partner drugs such as MQ or piperaquine [19, 36]. Efficacy of AS-MQ has not previously been described in CQ-resistant *P. vivax*. However, the successful use of MQ monotherapy against CQ-resistant *P. vivax* has been reported in a prospective study from Indonesia [37]. Our study provides further evidence for the efficacy of AS-MQ against CQ-resistant *P. vivax,* including those patients who had recently failed CQ treatment. The efficacy of MQ in areas such as Indonesia and Malaysia with ongoing widespread CQ use may be aided by competitive drug selection pressure occurring between CQ and MQ. In such regions, the *pvmdr1* mutations associated with *P. vivax* CQ resistance potentially increase the parasites' susceptibility to MQ [38].

Patients treated with AS-MQ exhibited a faster early therapeutic response, with significantly shorter parasite and fever clearance times compared with CQ. This benefit was apparent even in those patients with no subsequent recurrence. The higher rates of both ring and trophozoite clearance with AS-MQ are also consistent with previous in vitro drug susceptibility testing, demonstrating relatively lower inhibitory concentrations (IC_50_) of AS compared with CQ [39]. AS-MQ also demonstrated better hematological outcomes, a 4-fold lower risk of anemia at day 28, and lower risk of gametocyte carriage in the AS-MQ arm and thus of onward transmission.

The faster parasite clearance and reduced readmission to the hospital also resulted in significant cost-effectiveness with AS-MQ, highlighted by approximately 1.6-fold lower bed-occupancy rates. The Malaysian Ministry of Health estimates the subsidized cost of an inpatient with uncomplicated malaria to be $66/day, with the price of the full course of CQ estimated at $0.38 and generic AS-MQ $2.13 (personal communication, Ministry of Health, Malaysia). Based on our study's bed-occupancy data, this gives a minimum cost-saving difference for hospital inpatient days of $163 218 per 1000 patients treated with ACT, which far exceeds the higher medication cost of $1750 per 1000 patients.

The interpretation of CQ efficacy in *P. vivax* is challenging [40]. Confirmation of resistance requires demonstration of parasite growth at drug concentrations that would normally kill or inhibit further development. The recurrent appearance of *P. vivax* in the peripheral blood smear within 28 days of CQ treatment is highly indicative of resistance. However, recurrent infections can be due to inadequate drug absorption or reinfection in the presence of low blood CQ concentrations following rapid drug metabolism and elimination. All patients treated with CQ in our study had observed administration with correct dosage based on body weight and confirmed therapeutic plasma CQ concentrations at day 7. Furthermore, all recurrent infections were homologous, which is in keeping with them being true recrudescences, although the possibility of homologous CQ-sensitive relapses cannot be excluded in those recurring in the presence of CQ + DCQ concentrations below the MEC. At the time of recurrence, 9 of 20 patients assessed had plasma CQ + DCQ concentrations in excess of 15 ng/mL (range, 19.7–to 120.5), demonstrating parasite growth in the presence of adequate drug concentrations. By definition, these parasites are CQ resistant.

Limitations of the study included the relatively small number of patients enrolled in each arm. Furthermore, using the standard commercially sourced medications, it was not possible to blind research staff to treatment allocation after the initial randomization. Importantly, however, the primary and most secondary outcome measures were determined by microscopy, with study microscopists blinded to treatment allocation. Primaquine administration was delayed to day 28 since its blood-stage activity limits can confound the interpretation of CQ efficacy [41]. However, the administration of a 14-day treatment course of primaquine started on day 28 may have contributed to the lack of any *P. vivax* recurrences between day 28 and day 42.

### Conclusions

This prospective study represents the first antimalarial efficacy study of *P. vivax* in Malaysia. It demonstrates hitherto undetected high-grade CQ resistance and associated high risk of clinical failure to CQ treatment. Despite increasing reports of CQ resistance, CQ remains the first-line therapy for *P. vivax* in most vivax-endemic countries. The identification of high levels of CQ resistance in Malaysia highlights that CQ efficacy cannot be assumed and supports the need for ongoing evaluation of its efficacy in vivax-endemic regions [2, 22]. AS-MQ was highly efficacious in treating the CQ-resistant *P. vivax* in the study area. Additional clinical and public health advantages of AS-MQ treatment included faster parasite and fever clearance, earlier hospital discharge, lower bed occupancy rates, decreased anemia and morbidity, and lower transmission risk. Our findings support a switch to an ACT for vivax malaria in this region as recommended by the WHO if >10% resistance is documented [22]. A unified ACT blood-stage treatment guideline for all *Plasmodium* spp. would provide excellent efficacy for the artemisinin-sensitive *P. falciparum* and *P. knowlesi* and CQ-resistant *P. vivax* found in co-endemic areas such as Malaysia. Further therapeutic efficacy trials of alternative ACTs for uncomplicated *P. vivax* malaria addressing both safety and efficacy are warranted.

## Supplementary Data

Supplementary materials are available at http://cid.oxfordjournals.org. Consisting of data provided by the author to benefit the reader, the posted materials are not copyedited and are the sole responsibility of the author, so questions or comments should be addressed to the author.

Supplementary Data
