# Supplementary material for: Efficacy of Artesunate-mefloquine for Chloroquine-resistant Plasmodium vivax Malaria in Malaysia: An Open-label, Randomized, Controlled Trial
Source: Clin Infect Dis. 2016 May 12;62(11):1403–11. doi: 10.1093/cid/ciw121 (PMC4872287; doi:10.1093/cid/ciw121)
Supplement: Supplementary Data [file supp_62_11_1403__index.html]

Efficacy of Artesunate-mefloquine for Chloroquine-resistant Plasmodium vivax Malaria in Malaysia: An Open-label, Randomized, Controlled Trial — Efficacy of Artesunate-mefloquine for Chloroquine-resistant Plasmodium vivax Malaria in Malaysia: An Open-label, Randomized, Controlled Trial — Supplementary Data 

# Efficacy of Artesunate-mefloquine for Chloroquine-resistant *Plasmodium vivax* Malaria in Malaysia: An Open-label, Randomized, Controlled Trial

## Supplementary Data

Supplementary Data

- Supplementary Data - Docx file
