# Supplementary material for: Efficacy of Artesunate-mefloquine for Chloroquine-resistant Plasmodium vivax Malaria in Malaysia: An Open-label, Randomized, Controlled Trial
Source: Clin Infect Dis. 2016 May 12;62(11):1403–11. doi: 10.1093/cid/ciw121 (PMC4872287; doi:10.1093/cid/ciw121)
Supplement: Supplementary Data [file supp_ciw121_ciw121supp.docx]

**Supplemental material:**

**“Efficacy of artesunate-mefloquine against high-grade chloroquine-resistant *Plasmodium vivax* malaria in Malaysia: an open-label randomised controlled trial”**

***Corresponding author:**

Dr Matthew Grigg

Global and Tropical Health Division, Menzies School of Health Research

PO Box 41096, Casuarina, Darwin 0811, Northern Territory, Australia

[matthew.grigg@menzies.edu.au](mailto:matthew.grigg@menzies.edu.au)

**1. LC/MS/MS measurement of Chloroquine (CQ) and Desethylchloroquine (DCQ)**

***Methods:***

Plasma CQ and DCQ concentrations were measured by liquid chromatography-tandem mass spectrometry (LC/MS/MS). Chromatographic analysis was conducted with a Prominence UFLC-XR liquid chromatography system (Shimadzu, Japan) linked to an AB Sciex 4000 Q Trap mass spectrometer (Applied Biosystems) fitted with an electrospray interface operated in the positive-ion mode. The reconstituted sample was separated on a Luna PFP-2 analytical column (5µ, 50 mm x 4.6 mm I.D., Phenomenex®) with a Luna PFP2 guard column (4 x 2 mm I.D.) maintained at 30˚C in a column oven with a mobile phase consisting of an isocratic mix in the ratio 50:50 of Pump A: 2 mM perfluoro-octanoic acid and Pump B: acetonitrile pumped at a combined flow rate of 0.5 mL/min.

Plasma calibration standards of CQ and DCQ were prepared by spiking acid citrate dextrose anticoagulated human plasma to produce a 2,000 ng/mL solution of CQ and DCQ, with further dilutions made in plasma to produce calibrators over the range of 0.5–1,000 ng/mL. Aliquots of 250 µL were dispensed into 1.8 mL cryo tubes and stored at -80ºC.

Liquid-liquid extraction was applied for sample preparation. Briefly, to a polypropylene microcentrifuge tube were added CQ and DCQ spiked plasma or patient plasma (50 µL), chloroquine analog (CQa) (internal standard, 25 µL of a 1 ng/mL solution in 50% acetonitrile), 100 µL of 30% ammonium solution and 1 mL of methyl-*tert*-butyl ether. The sample was mixed for 10 min and centrifuged at 20,000 *g* for 15 min at 4˚C. Supernatant (800 μL) was transferred to a 1.8 mL microcentrifuge tube and the contents evaporated to dryness at 40ºC under a stream of instrument grade air. Mobile phase (150 μL) was added to the residue in each tube and mixed for 1 min. The contents of the tubes (2 µL) was injected onto the column.

Quantification was performed using multiple reaction monitoring (MRM) of the ion transitions of 320.199 to 247.1 and 142.2 *m*/*z* for CQ, 292.189 to 179.0 and 114.3 *m*/*z* for DCQ and 334.192 to 261.1 and 142.2 *m*/*z* for CQa. Quadratic regression for the calibration standards (range 0.5 to 1,000 ng/mL) with a weighting factor of 1/x^2^ yielded a correlation coefficient of >0.992 for both analytes. The retention times of CQ, DCQ and CQa were at 0.91, 0.91 and 1.08 min, respectively. The lower limit of quantification was 0.5 ng/mL with 50 µL of plasma for both CQ and DCQ.

The interassay precision of analysis (percent coefficient of variation [CV]) for CQ of 8.5% at 0.5 ng/mL, 4.8% at 100 ng/mL and 5.3% at 1,000 ng/mL (n≥11), with an inaccuracy of ≤1.5%. Corresponding CV values for DCQ were 8.5%, 7.0% and 6.0%, respectively, with an inaccuracy of ≤1.1%.

***Table A.*** *Composite* *plasma chloroquine (CQ) and desethylchloroquine (DCQ) concentrations*

| Variable | **Treatment failure by day 28**  n=26* | **No treatment failure by day 28**  n=16 | **P value** |
| --- | --- | --- | --- |
| Day 7 |  |  |  |
| Sample obtained n (%) | 22 (88) | 15 (94) | 0.571 |
| Median concentration ng/mL (IQR) | 44.2 (26.9-73.0) | 51.7 (37.5-72.0) | 0.194 |
| Median ratio CQ : DCQ (IQR) | 2.0 (1.7-2.3) | 2.1 (1.5-2.4) | 0.756 |
|  |  |  |  |
| Day of parasite recurrence |  |  |  |
| Sample obtained n (%) | 20 (80) | - |  |
| Median day of recurrence (IQR) | 22 (13-28) | - |  |
| Median concentration ng/mL (IQR) | 8.6 (5.1-27.2) | - |  |
| Above minimal effective  concentration (>15ng/mL) n/N (%) | 9/20 (45) | - |  |

*One patient with early treatment failure did not complete CQ course and their drug concentrations were not included in the analysis

**2. Genotyping**

***Molecular Processing***

With the exception of one sample pair, where only a dried blood spot was available for the recurrent infection, molecular processing was undertaken on venous blood samples collected into in EDTA tubes. Between 200 ul and 1 ml blood was extracted using the QIAamp blood mini or midi kits (Qiagen) according to the manufacturer’s protocols. Molecular confirmation of *Plasmodium* spp. was undertaken using the method described by Padley et al.(1). *P. vivax* genotyping was undertaken at nine previously described short tandem repeat (STR) markers: *Pv3.27, msp1F3, MS1, MS5, MS8, MS10, MS12, MS16* and *MS20*(2,3). These markers are included in a consensus panel selected by partners within the Vivax Working Group of the Asia Pacific Malaria Elimination Network. The *Pv3.27, MS16* and *msp1F3* loci were amplified using methods described elsewhere(4). The protocol for the remaining loci and the details of the primer sequences and chromosomal locations for each marker have been provided previously(4,5). The labelled PCR products were sized by denaturing capillary electrophoresis on an ABI 3100 Genetic Analyzer with GeneScan LIZ-600 (Applied Biosystems) internal size standards. Genotype calling was undertaken using GeneMapper Version 4.0. To reduce potential artefacts, an arbitrary fluorescent intensity threshold of 100 rfu was applied for peak detection. All electropherogram traces were additionally inspected manually.

***Analysis of PCR adjustment***

The genotype profiles of pairs of day 0 and recurrent samples were compared at all 9 loci to determine whether the recurrent infections were homologous (same) or heterologous (different) to the day 0 infections. Recurrent samples were classified as homologous (recrudescence/relapse) if at least one allele was shared with the day 0 sample at each locus investigated, and heterologous (re-infection/relapse) if no alleles were shared with the day 0 sample at 1 or more loci. Recurrence outcomes were defined as indeterminate if a sample pair exhibited no informative data at all loci investigated. Informative data from a minimum of 3 loci was required to call a homologous recurrence event. For calculation of the PCR-adjusted cure rate, homologous recurrences were classified as recrudescences (although these may include homologous relapses) and heterologous recurrences were classified as re-infections or heterologous relapses.

**3. Adverse events summary**

| System | Adverse event | AS-MQ  n* (%) | CQ  n (%) | P-value |
| --- | --- | --- | --- | --- |
| Gastrointestinal | Vomiting | 30 (55) | 23 (48) | 0.444 |
|  | Abdominal pain | 13 (24) | 12 (25) | 0.914 |
|  | Diarrhoea | 5 (9) | 3 (6) | 0.573 |
|  | *Any of above* | 37 (69) | 29 (60) | 0.393 |
| Neurological | Dizziness | 40 (74) | 39 (81) | 0.387 |
|  | Headache | 49 (91) | 44 (92) | 0.869 |
|  | Vision/hearing | 7 (13) | 7 (15) | 0.812 |
|  | *Any of above* | 45 (94) | 51 (94) | 0.882 |
| Skin | Rash/itch | 4 (7) | 2 (4) | 0.487 |
| Respiratory | Cough | 15 (28) | 18 (38) | 0.295 |
|  | Shortness of breath | 12 (22) | 4 (8) | 0.054 |
|  | *Any of above* | 19 (40) | 22 (41) | 0.905 |
| Retreatment | Vomiting < 1 hour post medication** | 0 | 2 (8) | 0.224 |
| Rescue treatment |  | 0 | 4 (8) | **0.048** |

*n = number of patients

**Both patients retreated with CQ had an adequate clinical and parasitological treatment outcome at day 28

***References:***

1. Padley D, Moody AH, Chiodini PL, Saldanha J. Use of a rapid, single-round, multiplex PCR to detect malarial parasites and identify the species present. Ann Trop Med Parasitol. 2003 Mar;97(2):131–7.

2. Karunaweera ND, Ferreira MU, Munasinghe A, Barnwell JW, Collins WE, King CL, et al. Extensive microsatellite diversity in the human malaria parasite Plasmodium vivax. Gene. 2008 Feb;410(1):105–12.

3. Koepfli C, Müeller I, Marfurt J, Goroti M, Sie A, Oa O, et al. Evaluation of Plasmodium vivax Genotyping Markers for Molecular Monitoring in Clinical Trials. J Infect Dis. 2009 Apr;199(7):1074–80.

4. Abdullah NR, Barber BE, William T, Norahmad NA, Satsu UR, Muniandy PK, et al. Plasmodium vivax Population Structure and Transmission Dynamics in Sabah Malaysia. Braga ÉM, editor. PLoS One. 2013 Dec 17;8(12):e82553.

5. Gunawardena S, Karunaweera ND, Ferreira MU, Phone-Kyaw M, Pollack RJ, Alifrangis M, et al. Geographic structure of Plasmodium vivax: microsatellite analysis of parasite populations from Sri Lanka, Myanmar, and Ethiopia. Am J Trop Med Hyg. 2010 Feb;82(2):235–42.
